# Sero-Molecular Epidemiology of Japanese Encephalitis in Zhejiang, an Eastern Province of China

**DOI:** 10.1371/journal.pntd.0004936

**Published:** 2016-08-25

**Authors:** Jin-ren Pan, Ju-ying Yan, Jia-yue Zhou, Xue-wen Tang, Han-qing He, Rong-hui Xie, Hai-yan Mao, Yan-jun Zhang, Shu-yun Xie

**Affiliations:** Zhejiang Provincial Center for Disease Control and Prevention, Hangzhou, People’s Republic of China; Santa Fe Institute, UNITED STATES

## Abstract

**Background:**

Sporadic Japanese encephalitis (JE) cases still have been reported in Zhejiang Province in recent years, and concerns about vaccine cross-protection and population-level immunity have been raised off and on within the public health sphere. Genotype I (GI) has replaced GIII as the dominant genotype in Asian countries during the past few decades, which caused considerable concerns about the potential change of epidemiology characteristics and the vaccine effectiveness. The aim of this study was to investigate the prevalence of JE neutralizing antibody and its waning antibody trend after live attenuated JE vaccine immunization. Additionally, this study analyzed the molecular characteristics of the E gene of Zhejiang Japanese encephalitis virus (JEV) strains, and established genetic relationships with other JEV strains.

**Methodology/Principal Findings:**

A total of 570 serum specimens were sampled from community population aged from 0 to 92 years old in Xianju county of Zhejiang Province in 2013–2014. Microseroneutralization test results were analyzed to estimate the population immunity and to observe antibody dynamics in vaccinated children. E genes of 28 JEV strains isolated in Zhejiang Province were sequenced for phylogenetic tree construction and molecular characteristics analysis with other selected strains. Positive JE neutralizing antibody rates were higher in residents ≥35 years old (81%~98%) and lower in residents <35 years old (0~57%). 7 or 8 years after the 2^nd^ live attenuated vaccine dose, the antibodies against for 4 different strains with microseroneutralization test were decreased by 55%~73% on seropositive rates and by 25%~38% on GMTs respectively. JEV strains isolated in recent years were all grouped into GI, while those isolated in the 1980s belonged to GIII. On important amino acid sites related to antigenicity, there was no divergence between the Zhejiang JE virus strains and the vaccine strain (SA14-14-2).

**Conclusion/Significances:**

JE neutralizing antibody positive rates increase in age ≥10 years old population, likely reflecting natural infection or natural boosting of immunity through exposure to wild virus. JE seropositivity rates were quite low in <35 years old age groups in Zhejiang Province. Waning of neutralizing antibody after live attenuated vaccine immunization was observed, but the clinical significance should be further investigated. Both the peripheral antibody response and genetic characterization indicate that current live attenuated JE vaccine conferred equal neutralizing potency against GI or GIII of wild strains. GI has replaced GIII as the dominant genotype in Zhejiang in the past few decades. Although the chance of exposure to wild JE virus has reduced, the virus still circulates in nature; therefore, it is necessary to implement immunization program for children continually and to conduct surveillance activity periodically.

## Author Summary

Japanese encephalitis (JE) remains one of the most significant public health problems in Asia and the Western Pacific region. A JE viral infection can cause death and severe sequelae. Vaccination is the most effective method for preventing JE currently. After decades of routine vaccination, the number of JE cases declined considerably in Zhejiang Province, China. However, emergence of genotype I of JE as the most common genotype in China in recent decades has become a major public health problem. As all the currently available vaccines are derived from genotype III strains, the circulations of another genotype have caused considerable concerns about vaccine effectiveness. In this study, we found that population immunity against JE was quite low in children and adolescents. Waning of JE neutralizing antibody after JE immunization was observed. Therefore, issues about duration of protection and booster dose necessity need further research. On the bright side, evidence shows that the JE vaccine currently used is effective for both genotype I and III of wild viruses. Although clinical JE cases have reduced, the virus is still spreading in nature; therefore, we encourage children and other high-risk groups to adhere to the immunization program continuously.

## Introduction

Japanese encephalitis (JE) is a common mosquito-borne viral encephalitis disease and it is prevalent in Asia, the Western Pacific, and northern Australia. It is estimated that approximately 67,900 JE cases occur worldwide annually, with a fatality rate range from 20% to 30%. Though reported cases have decreased dramatically due to immunization programs, improved living conditions and avoiding animal hosts, as an enzootic cycle disease, JE will remain a prominent public health problem in the Asian-Pacific region [1,2].

JE is caused by the Japanese encephalitis virus (JEV). The 1500-nt envelope (E) protein gene was suggested to provide reliable information reflecting the broad geographical and temporal relationships of JEV [3,4]. Based on the E gene, JEV can be divided into five genotypes [5] and the different genotypes have certain regional distribution features [6]. Genotype I (GI) and III (GIII) are mostly associated with epidemic diseases in temperate regions of Asia [7]. Three JEV genotypes have been isolated in China so far. The dominant genotypes were GI and GIII, only one strain of genotype V was reported to have been isolated in Tibet in 2009 [5,8]. As all the currently available vaccines are derived from GIII strains, circulation of other genotypes has caused theoretical concern about the vaccine effectiveness [9].

**Fig 1:**
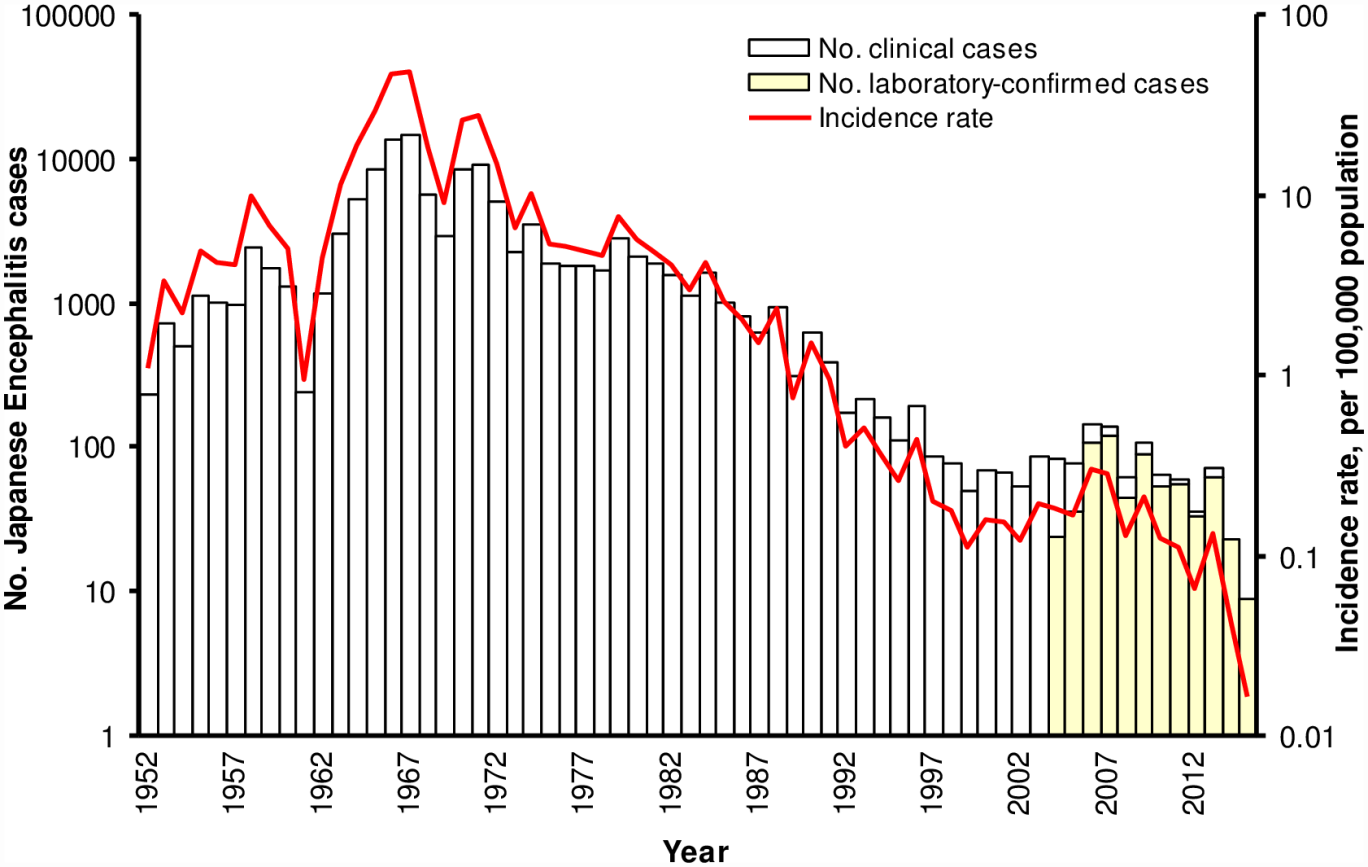
Morbidity of Japanese encephalitis in Zhejiang Province, China, 1952–2014. Data from the National Notifiable Diseases Reporting System, a hospital-based passive reporting system. Laboratory testing method was established to examine reported cases since 2004 in Zhejiang Province.

Zhejiang is an eastern coastal province in China that situated in the subtropical climate area. It has an area of 101,800 km^2^ and a population of 54.43 million (2010 census data). According to the detailed morbidity data since 1952, JE was epidemic in the 1960s and 1970s in Zhejiang Province. The epidemic peaked in 1967, with 14,597 cases of JE reported, representing an annual incidence rate of 47.5/100,000. Through a combination of vaccination, improved housing and socioeconomic conditions, environmental management, and vector-control efforts [10], the morbidity rate has decreased sharply after 1970s. Since 1990, the incidence rate was less than 1/100,000, and it further dropped to less than 0.5/100,000 in the past 5 years with only dozen sporadic cases reported annually (Fig 1). Universal vaccination with an inactivated JE vaccine (P3 strain) on children under 10 years old was launched in Zhejiang Province in 1970s. JE vaccine was introduced in routine immunization program in 1986 and a live attenuated vaccine (SA14-14-2 strain) replaced the inactivated vaccine quickly in Zhejiang Province since 1999. Currently, two doses of the live attenuated vaccine are required in national immunization program, the first at 8 months and the second as a booster dose at 2 years old [10].

Based on the pathogenic surveillance activity, dozen of JEV strains have been isolated in Zhejiang Province in 1980s and between 2007–2014. Additionally, a serosurvey was conducted in community population in 2013–2014 in Zhejiang Province. Although several population-based seroprevalence surveys of JE had been reported in other regions, most of them only used a single viral strain in neutralizing antibody test and lacked a refined division of age groups among children. To our knowledge, an exhaustive seromolecular epidemiology of JE study in Zhejiang Province has not been reported. In this study, we collected serum samples from 570 subjects in 2013–2014 serosurvey to investigate the prevalence of JE neutralizing antibody and to elucidate the waning antibody trend after live attenuated JE vaccine. Additionally, we analyzed the molecular characteristics of the E gene of 28 JEV strains isolated in Zhejiang Province and established their genetic relationships with other JEV strains.

## Materials and Methods

### Ethics Statement

This work was approved by the ethics committee of the Zhejiang Provincial Center for Disease Control and Prevention and was conducted in accordance with Good Clinical Practice guidelines. Written informed consents were obtained from all participants or guardians (for children ≤18 years) prior to enrollment in the study. The subjects’ names were not disclosed to the authors.

### Specimen Collection

**Fig 2:**
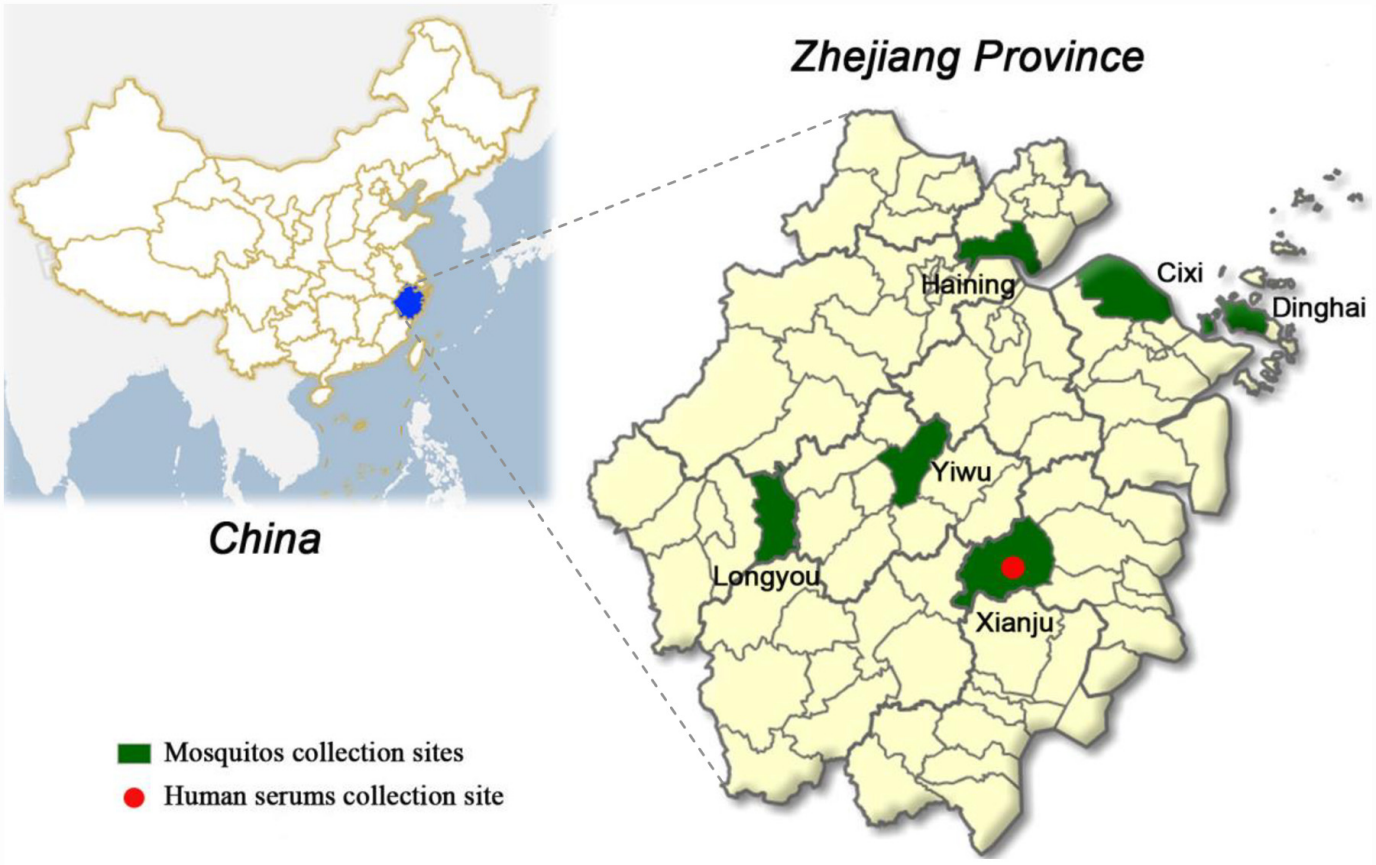
The counties of sample collection site in Zhejiang Province, China. Maps were created by an online map service system (http://www.dituhui.com/).

Fig 2 shows the geographical position of Zhejiang Province and the samples collection sites (county) in the present study.

#### Human serum samples

A cross-sectional serosurvey was carried out in Xianju county of Zhejiang Province in 2013–2014. Sample size for frequency in a population was calculated by OpenEpi 3.03 version (http://www.openepi.com). With the anticipated frequency as 80%, confidence limits as ±5%, design effect for cluster surveys as 2, the sample size with 95% confidence level was 492. An age-stratified cluster sampling method was used in participants’ enrollment. The cluster was defined as an institution or a crowd such as a class in a school or kindergarten, a nursing home, a residential area, children for routine immunization visit in a clinic, etc. The participants then divided into 14 age groups mainly according to the JE vaccine immunization program and the risk of disease exposure. In short, the younger of the participants sampled, the shorter of the age interval categorized. A total of 570 serum specimens were sampled from community population aged from 0 to 92 years old (Table 1). Participants were requested to give a blood sample and to fill out a self-administered questionnaire. Sera were transferred in ice boxes and stored at -80°C until processed.

**Table 1:** Demographic characteristics of subjects.

	*N*	%
**Gender** (*N* = 570)		
Male	307	53.9
Female	263	46.1
**Age** (*N* = 570)		
0–2 M	49	8.6
3–5 M	19	3.3
6–8 M	21	3.7
9–11 M	24	4.2
1 Y	38	6.7
2 Y	37	6.5
3–4 Y	44	7.7
5–6 Y	53	9.3
7–9 Y	64	11.2
10–12 Y	39	6.8
13–14 Y	55	9.6
15–34 Y	44	7.7
35–59 Y	37	6.5
≥60 Y	46	8.1
**Years from Booster in Children** (*N* = 185)		
0	15	8.1
1–2	34	18.4
3–4	48	25.9
5–6	46	24.9
7–8	42	22.7

#### Mosquito samples

Mosquitoes were collected in pig-pens or cattle sheds proximal to human activity sites and in private houses in rural areas (Dinghai, Yiwu, Xianju, Cixi, Haining and Longyou county of Zhejiang Province) during June-August in the given year. Hand-held aspirators and lamp-traps were used to collect mosquitoes in the evening. The periods of mosquito collection were divided into two stages, 1982–1983 and 2007–2014. A total of 49,808 mosquitoes were collected, with the dominant species being *Culex tritaeniorhynchus* (Table 2). Specimens were placed in liquid nitrogen on transportation and stored at -80°C until processed for virus isolation.

**Table 2:** Mosquitoes collected for Japanese encephalitis study in Zhejiang Province, China, 1982–3 and 2007–2014.

Year	No. of Individuals (%)				Total
	*Culex tritaeniorhynchus*	*Culex pipiens pallens*	*Anopheles sinensis*	Others	
1982	816 (56.3)	259 (17.9)	252 (17.4)	123 (8.5)	1450
1983	742 (42.7)	320 (18.4)	519 (29.9)	157 (9.0)	1738
2007	4146 (38.9)	2427 (22.8)	3341 (31.3)	748 (7.0)	10662
2009	5257 (81.2)	545 (8.4)	512 (7.9)	157 (2.4)	6471
2010	4953 (69.3)	356 (5.0)	589 (8.2)	1254 (17.5)	7152
2012	4290 (70.2)	131 (2.1)	1224 (20.0)	465 (7.6)	6110
2013	2289 (41.3)	247 (4.5)	2786 (50.3)	218 (3.9)	5540
2014	6069 (56.8)	850 (8.0)	2308 (21.6)	1458 (13.6)	10685
Total	28562 (57.3)	5135 (10.3)	11531 (23.2)	4580 (9.2)	49808

### Japanese Encephalitis Incidence Rates

JE cases have been a legally reportable communicable disease in China since 1951[5]. When physicians reported a suspected case, the local county’s Center for Disease Control and Prevention staff would carry out the case investigation and collect serum or cerebrospinal fluid specimens for diagnosis verification in recent years. For calculation of yearly average incidence rates, the onset day between 2010 and 2014 of JE cases were exported from the National Notifiable Diseases Reporting System (NNDRS), an internet-based real-time case reporting system, which was established in 2004. Non-Zhejiang Province citizen cases were excluded in calculating the yearly average incidence rates between 2010–2014.

### Microseroneutralization Assay

Microseroneutralization tests were conducted under biosafety level 2 conditions in the JE laboratory of the Zhejiang Provincial Center for Disease Control and Prevention, a member of the National Reference JE Laboratory. The procedure has been described elsewhere in detail [11,12]. Briefly, serum samples were heated for 30 min at 56°C and titrated with 6 dilutions (1:10; 1:20; 1:40; 1:80; 1:160; 1:320). An equal volume of JEV (50% tissue culture infective dose [TCID 50] in 100 μL) was added to all specimen sera, and the plates were incubated for 1.5 h at 37°C. Four strains of JEV, i.e. P3 (GIII), ZJ83-8 (GIII), ZJ10-7 (GI) and ZJ13-3 (GI), were neutralized by each serum sample. The P3 strain was obtained from the National Institute for Food and Drug Control, China, and the other strains were isolated from mosquito samples in Zhejiang Province in 1983, 2010, and 2013, respectively. The mixture was added to a monolayer of the cell line BHK-21 (Baby Hamster Kidney) in a 96-well plate, and the plate was then incubated in a 5% CO_2_ humidity chamber at 37°C. Observation of cytopathic effects (CPE) began at 48 h later, and neutralization titers were determined 7 days later.

### Phylogenetic Analysis

#### Virus isolation

Mosquito samples were identified according to morphological characteristics and pooled by different collection sites, dates of collection, and species (every 50–100 female individuals per pool). Mosquito pools were milled thoroughly followed by centrifugation (12,000 r/min, 30 min). The supernatants were then inoculated onto monolayer BHK-21 cells or C6/36 (*Aedes albopictus*) cells. Culture mediums were then harvested after the cytopathic effects (75%-100%) had developed. The viruses were then confirmed by RT-PCR and E gene sequencing methods. In the 805 pools, 69 (8.6%) JEV positive pools were harvested.

#### Sequencing of E genes

Viral RNA was extracted from the JEV-infected culture medium using the RNeasy mini kit (Qiagen, Hilden, Germany). The E gene was amplified with primers JE955f (5′-TGYTGGTCGCTCCGGCTTA) and JE2536r (5′-AAGATGCCACTTCCACAYCTC) [13]. Reverse transcription–polymerase chain reaction (RT-PCR) was carried out by TaKaRa One Step RNA PCR Kit (TaKaRa Bio Inc., Dalian, China). The mixture was incubated at 50°C for 30 min, 94°C for 2 min, and 40 cycles at 94°C for 30 s, 50°C for 30 s, 72°C for 2 min, and elongation at 72°C for 8 min. Amplified products were examined by agarose gel electrophoresis (1.5%). After purification of the amplicons, E gene sequences were determined by Sangon Biotech Co., Ltd (Shanghai, China). A total of 28 strains of JEV were selected for sequencing.

#### Phylogenetic tree construction

Nucleotide sequences were edited and aligned by using DNAman 7 (Lynnon Biosoft). Phylogenetic and molecular evolutionary analyses were performed using the MEGA 6.2 software (Tamura, Stecher, Peterson, Filipski, and Kumar 2013). The phylogenetic tree was constructed by using the neighbour-joining method, and genetic distances were calculated according to Kimura's two-parameter method. The reliability of the tree was estimated by performing 1,000 bootstrap replications. Nucleotide sequences of the relevant strains of JEV were obtained from GenBank.

### Statistical Analysis

A serum sample with JE-neutralizing antibody ≥1:10 was considered as seropositive [14]. The chi-square test was used to assess the significance of seroprevalence between different strains or different age groups. The chi-square test for the linear trend was used to assess the association between seroprevalence with the elapsed years post-booster dose. Geometric mean titers (GMTs) of neutralizing antibody were calculated using a log transformation and were reported as back transformed titers. The results were presented in reciprocal form. Values below the detection threshold (1:10) were assigned half of the threshold value (1:5) in calculation. One-way analysis of variance was used to test the differences between strains, and multiple comparison tests were conducted by Bonferroni correction. Statistical analyses were performed by SPSS (Version 21.0, Chicago, USA) and two-sided *P*-values <0.05 were considered significant.

## Results

### GMTs and Seropositive Rates of JE Neutralizing Antibody

The overall GMTs of JE neutralizing antibody against 4 strains in participants were 8.4~11.3 with the seropositive rates 31.9%~42.8% (Table 3). Both of the GMTs (*F* = 12.47, *P*<0.001) and seropositive rates (χ^2^ = 15.91, *P* = 0.001) were significantly different between different strains. In multiple comparisons of GMTs among the 4 strains, the antibody against P3 strain was significantly higher than any other 3 wild virus strains, while there was no significant difference between ZJ83-8, ZJ10-7, and ZJ13-3 strains.

**Table 3:** Distribution of neutralizing antibody against different Japanese encephalitis virus strains.

Strain (Genotype)	No. of neutralizing antibody titer												Geometric mean titer (95% *CI*)	Seropositive rate (%)
	<10	10	15	20	30	40	60	80	120	160	240	320		
P3(GIII)	326	21	49	31	49	23	28	9	16	5	5	8	11.3 (10.3~12.3)	42.8
ZJ83-8(GIII)	388	30	43	28	33	20	10	4	7	2	2	3	8.4 (7.8~9.0)	31.9
ZJ10-7(GI)	373	31	47	38	29	16	16	10	4	3	0	3	8.7 (8.1~9.3)	34.6
ZJ13-3(GI)	365	36	58	34	33	15	14	5	3	1	2	4	8.7 (8.1~9.3)	36.0

**Fig 3:**
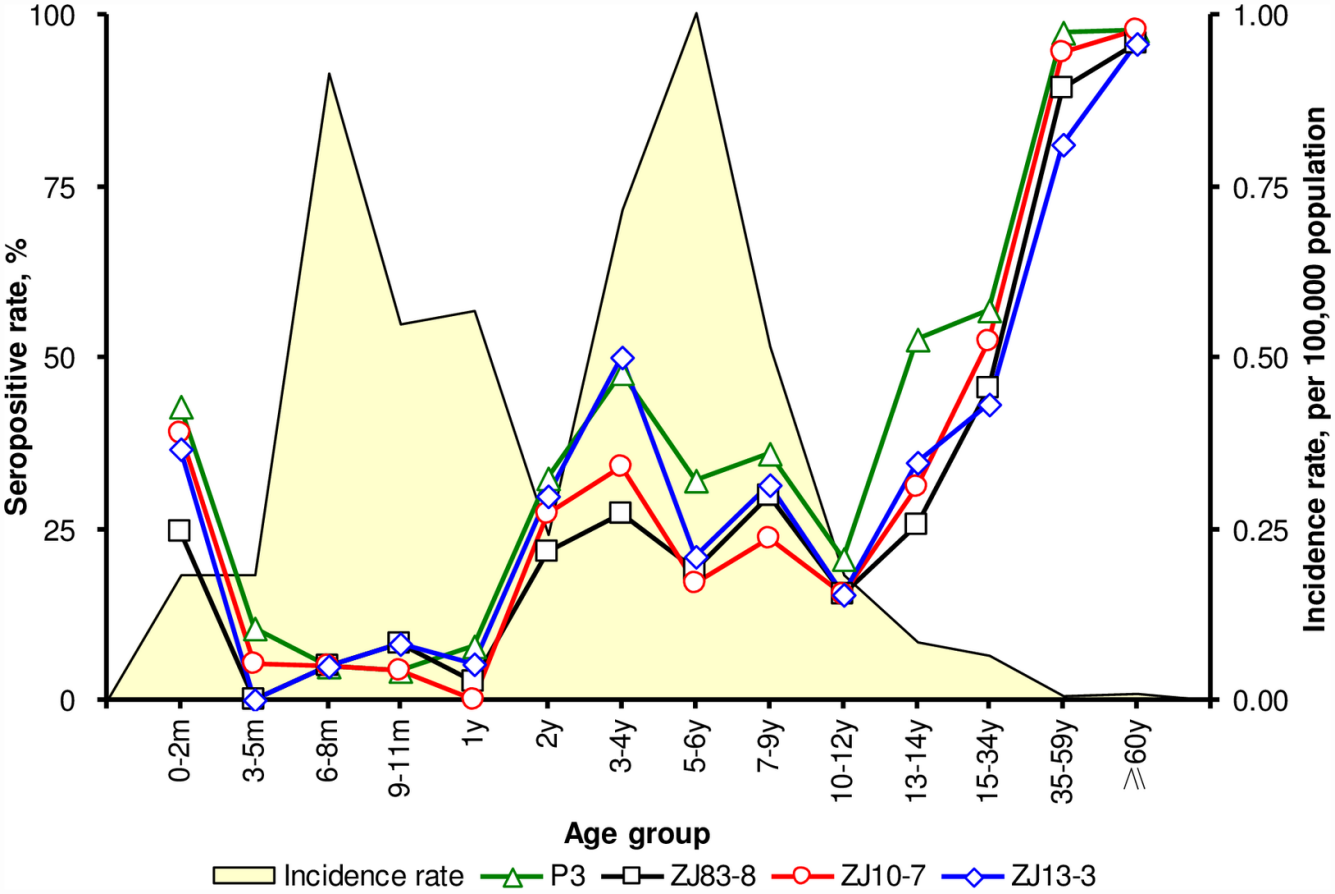
Age-specific seropositive rates of Japanese encephalitis neutralizing antibody and average incidence rates in Zhejiang Province, 2010–2014.

The seropositive rates of JE neutralizing antibody against 4 different strains had similar profiles (W-like curve) on age-group distributions (Fig 3). Starting from 24.5%~42.9% in <3 months old infants, the seropositive rates decreased to the lowest (0–10.5%) in 3 months-1 year old age group. The rates then increased moderately in the age group above 2 years old (the initial age of JE vaccine booster dose) followed by a dip in 10 years old age group. At last a dramatic rise could be seen in adults. In 35 years old age group, the rates increased to more than 80%, while in 60 years old age group all the rates ascended above 95%. The seropositive rates were statistically significant between <35 years old and ≥35 years old age groups (χ^2^ = 119.1, 165.5, 1624.4 and 119.3 for P3, ZJ83-8, ZJ10-7, ZJ13-3 strain, respectively, all *P*<0.001). To compare the yearly average incidence rates in Zhejiang Province between 2010 and 2014, 2 distinct age group peaks, i.e. 6 months and 5 years old age groups, were drawn out. The incidence rates dipped in the 7-year-old age group and older residents. The age-specific seropositive rates curve displayed quite contrary compared to the average incidence rates curve.

### Waning of Neutralizing Antibody after Booster Dose

**Fig 4:**
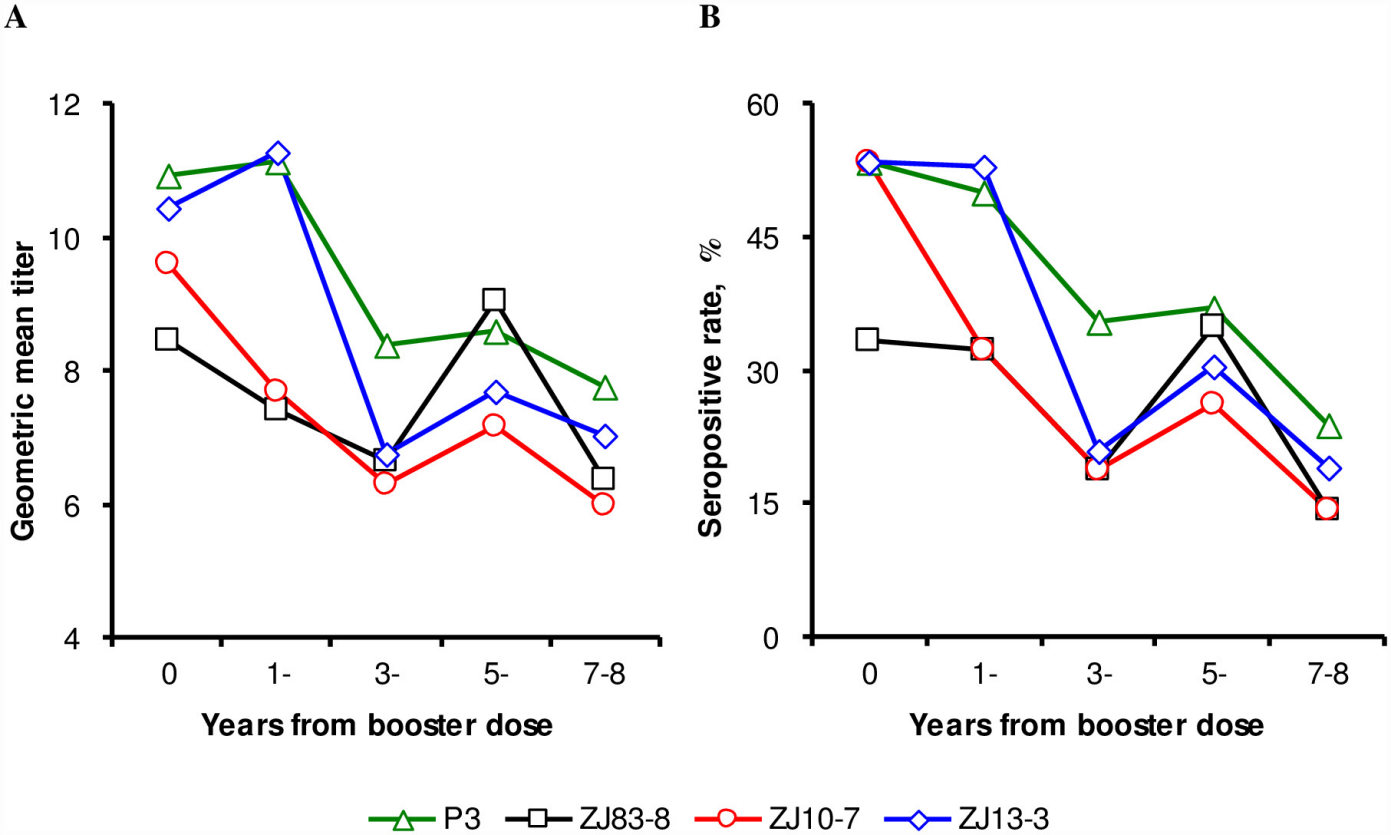
Geometric mean titers (A) and seropositive rates (B) of Japanese encephalitis neutralizing antibody on different year(s) post-booster dose.

Waning of JE neutralizing antibody after the 2^nd^ live attenuated vaccine dose was discovered in 185 child recipients. Generally, the longer of the booster dose elapsed, the lower of the GMTs and seropositive rates of JE-neutralizing antibody were (Fig 4). For example, the seropositive rate of neutralizing antibody against P3 strain declined from 53.3% to 23.8% 7–8 years after the year on booster dose, and the GMT declined from 10.93 (95%*CI*: 7.01~17.05) to 7.77 (95%*CI*: 5.75~10.48) as well. Except for the ZJ83-8 strain (χ^2^ for linear trend = 1.95, *P* = 0.163), the seropositive rates of the other 3 strains dropped with statistical significance (χ^2^ for linear trend = 6.67, 7.42, 10.31 and *P* = 0.010, 0.006, 0.001 for P3, ZJ10-7, ZJ13-3 strain, respectively). No significant differences (χ^2^ = 2.42, *P* = 0.298) on average seropositive rates between 3 wild strains (ZJ83-8, ZJ10-7 and ZJ13-3).

### Phylogenetic Analysis on E Genes

**Fig 5:**
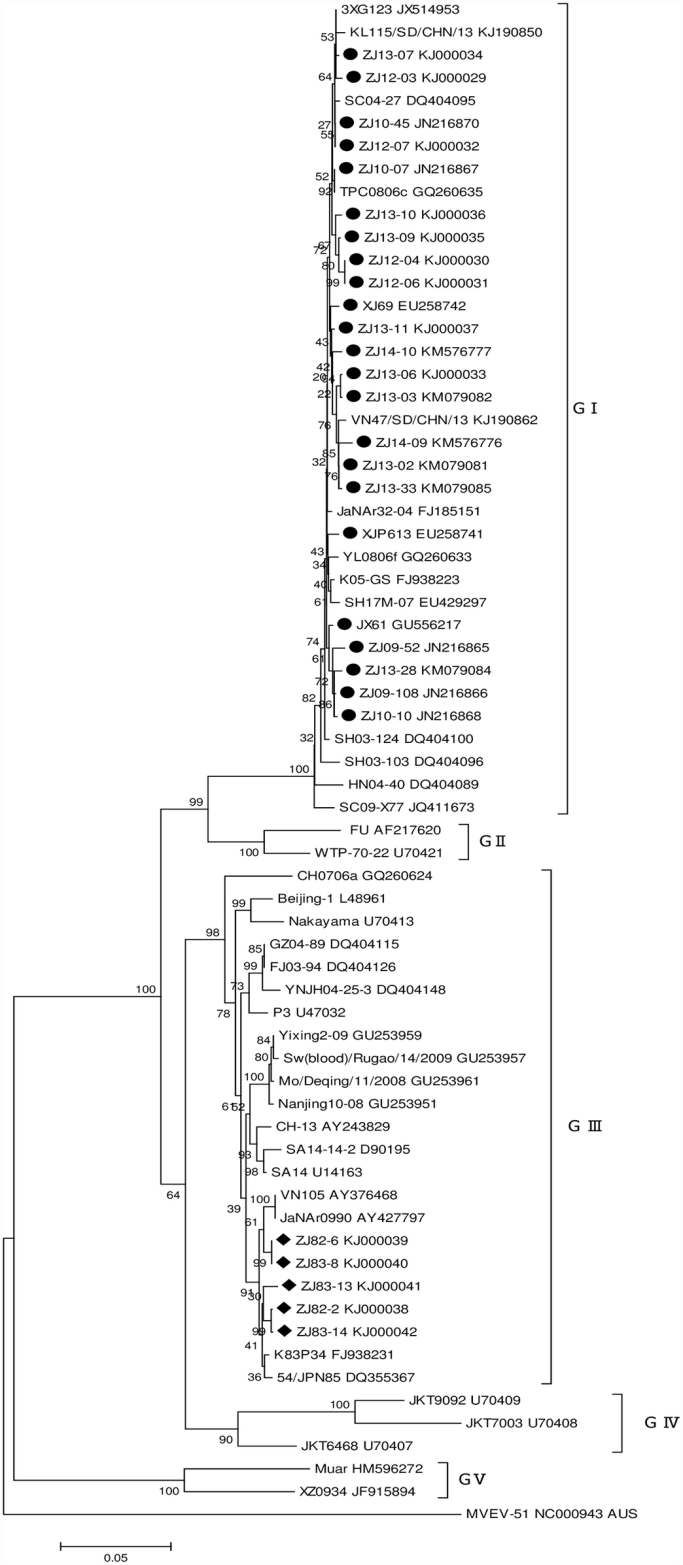
Phylogenetic tree on 1500-nt envelope gene of Japanese encephalitis virus strains. The sequences of Zhejiang strains isolated in 1982–1983 and 2007–2014 in this study are marked in black rhombus and circle, respectively. Phylogenetic analysis was performed by the neighbor-joining method using the MEGA 6.2 software package (www.megasoftware.net). Bootstrap probabilities of each node were calculated using 1000 replicates. Murray Valley encephalitis virus strain MVEV-51 was used as an outgroup. Scale bars indicate the number of nucleotide substitutions per site.

The 28 nucleotide sequences of E gene derived from Zhejiang Province (S2 Supplementary Data) were compared with selected strains from different isolation sites, times, and genotypes. In the phylogenetic tree, the Zhejiang strains were grouped into two genotypes (Fig 5), i.e. GI and GIII. The strains isolated in 2007–2014 were all clustered into GI, while those in 1982–1983 were all clustered into GIII. Zhejiang GI strains had a closer relationship with the China mainland JEV strains like SC04-27 (Sichuan, 2004), SH17M-07 (Shanghai, 2007), 3XG123 (Guangdong, 2011), and Taiwan strain TPC0806c (2008).

### Sequence Similarity Analysis on E Genes

E gene sequences of the Zhejiang JEV strains showed substantial genetic stability. The minimal sequence similarities were 88.0% and 98.8% at the nucleotide and amino acid sequence levels in 9 selected Zhejiang JEV strains. The nucleotide sequence divergences within the genotype were only 0.2%~1.5% (GI) and 0.9% (GIII). The nucleotide sequence divergence between the two genotypes was 11.7%~12.0%. Compared with the live attenuated vaccine strain (SA14-14-2), the nucleotide and amino acid sequence similarities were 87.7%~97.9% and 97.2%~98.0%, respectively (Table 4).

**Table 4:** Nucleotide and amino acid sequence similarities of envelope gene in selected Zhejiang Japanese encephalitis virus strains and vaccine strain (SA14-14-2).

Strain	Sequence similarity (%)									
	ZJ82-2	ZJ83-8	XJP613	JX67	ZJ09-52	ZJ10-45	ZJ12-04	ZJ13-07	ZJ14-10	SA14-14-2
ZJ82-2	-	99.1	88.1	88.1	88.0	88.3	88.1	88.3	88.2	97.8
ZJ83-8	100.0	-	88.1	88.1	88.1	88.3	88.1	88.3	88.2	97.7
XJP613	98.8	98.8	-	99.3	98.5	99.1	98.6	98.9	98.8	87.7
JX67	99.0	99.0	99.4	-	99.0	99.3	98.8	99.1	99.0	87.7
ZJ09-52	99.2	99.2	99.6	99.8	-	98.8	98.3	98.7	98.5	87.8
ZJ10-45	99.2	99.2	99.6	99.8	100.0	-	99.3	99.8	99.2	88.0
ZJ12-04	99.2	99.2	99.6	99.8	100.0	100.0	-	99.2	98.7	87.7
ZJ13-07	99.2	99.2	99.6	99.8	100.0	100.0	100.0	-	99.0	87.9
ZJ14-10	99.0	99.0	99.4	99.6	99.8	99.8	99.8	99.8	-	87.9
SA14-14-2	98.0	98.0	96.8	97.0	97.2	97.2	97.2	97.2	97.0	-

Note: The nucleotide similarities (%) are shown above the diagonal and the deduced amino acid similarities (%) are shown below the diagonal.

Eight amino acid residues of E protein (E107, E138, E176, E177, E264, E279, E315 and E439) were discovered as critical amino acid mutations in relation to virulence and virus attenuation [15]. To analyze these key amino acids, we compared the E protein of different genotypes of Zhejiang strains (ZJ82-2 and ZJ14-10) with the vaccine strain (SA14-14-2) and the known virulent strain (Beijing-1). No differences were found on the eight key amino acid residues among the two Zhejiang strains and Beijing-1 strain. On the contrary, these strains differed completely with SA14-14-2 strain on the eight residues. Therefore, it is deemed that both the GI and GIII Zhejiang strains possess typical characteristics of wild virulence (Table 5).

**Table 5:** Comparison of the amino acid residues of E protein between Zhejiang strains, SA14-14-2 and Beijing-1 strain.

Strain	Genotype	Amino acid residue of E protein							
		E107	E138	E176	E177	E264	E279	E315	E439
SA14-14-2	III	F	K	V	A	H	M	V	R
Beijing-1	III	L	E	I	T	Q	K	A	K
ZJ82-2	III	L	E	I	T	Q	K	A	K
ZJ14-10	I	L	E	I	T	Q	K	A	K

Note: Only two Japanese encephalitis virus strains obtained in Zhejiang are listed in the table because all Zhejiang strains with the same genotype share identical amino acids in these selected residues. A one-letter code is used for amino acid designation.

## Discussion

Our seroepidemiological findings indicate that JE vaccine immunization, waning of neutralizing antibody, and natural infection may influence the seroprevalence pattern in population. JE neutralizing antibody positive rates were higher in ≥35 years old age groups (81%~98%) in Zhejiang Province and lower in <35 years old age groups (0~57%). Seropositive rates fluctuated during the childhood. Beginning with the decay of maternally transferred antibody at birth, the rates reached the lowest in 3 months to 1 year old infants (0~10%). The rates increased to some extent after the booster dose (for 2 years old children) and then decreased gradually during the adolescent. The curves rose up dramatically in adults and in the aged, most of them should experience one or more natural infections in life. Our findings demonstrated quite the contrary between the age-specific seropositive rates and the average incidence rates. By the way, use of different JEV strains may have considerable influence on neutralizing antibody levels, so it is important to standardize the protocol for better comparability among different studies.

Heterogeneous seropositive rates were reported in different regions in recent years. The rate was 44% for 0–2 years old children in Jiangsu, a province adjacent to Zhejiang, China, where rates were above 70% for ≥3 years old children, 87% for 15–19, and 94% for ≥20 years old age groups [16]. In Shanxi Province, China, the positive rate was 95% for ≥10 years old age groups in high JE incidence rate prefectures, while the positive rate was only 22% in low incidence rate regions [17]. In two cities in Henan Province, China, the positive rates were 55% and 45% for 0–14 years old age groups, 98% and 49% for ≥15 years old age groups, respectively [18]. Based on a serum survey in Taiwan, researchers found that the cohort born between 1963 and 1975 exhibited the lowest positive rate (54%). The highest seropositive rate (86%) was observed in the cohort born before 1952 [19]. Investigation on the prevalence of neutralizing antibodies in high-risk age groups in South Korea, researchers found a very high seropositive rate (98%) in ≥30 years old residents [20]. Difference in rates between regions might owe to circulation intensity of JEV in local places, JE vaccination programs, or various neutralizing antibody test methods (including selection on JEV strains), etc. However, a similar feature was JEV seropositive rates increased with an increase of age group in high-risk areas.

The present work indicates that children immunized with 2 doses of live attenuated JE vaccine had equal immunogenicity against GI or GIII wild JEV strains, but the neutralizing potency was higher against P3 strain. Epidemiology surveillance data and animal experiments [21,22] also demonstrated that the JE vaccine could confer cross-protection, and our study may add valuable information on serum neutralizing test results. The result is also supported by surveillance data in Zhejiang Province (Fig 1). The genotype replacement events seem not bring about the irruption of new outbreaks. Waning of neutralizing antibody after live attenuated vaccine immunization was also observed in this work. The seropositive rates and GMTs decreased by 55%~73% and 25%~38%, respectively, for antibodies against the 4 different strains 7–8 years after the 2^nd^ dose. However, several studies pointed out that the protection correlated better with cellular immune responses than neutralizing antibody responses following live attenuated vaccine immunization [22,23]. Vaccine field trials also indicated that the duration of protection was quite long. A case-control study conducted in Nepal provided evidence that a single dose of the SA14-14-2 vaccine maintained a high protection (96%) for 5 years [24]. So, the clinical significance of waning antibody should be further investigated.

JEV E protein is closely related to virulence. Several existing studies have shown that virulence may be altered by mutations on critical residues. Researchers observed that a single amino acid substitution at E138 (Glu to Lys) was causally linked to attenuation [25]. As far as the eight amino acid residues of E protein critically related to the virulence, NO differences were found among GI, GIII of Zhejiang strains and the highly virulent strain (Beijing-1). Zhejiang JEV strains isolated in different periods all possessed typical characteristics of high virulence. Additionally, E protein is the dominant antigen in eliciting neutralizing antibody and protective immune responses. When comparing the important amino acid sites in relation to antibody-mediated virus neutralization [25–27], there was no divergence between Zhejiang JEV strains and SA14-14-2 strain. Therefore, genetic evidences supported the point of view that present live attenuated vaccine is still effective.

GIII strains had resulted in numerous JE epidemics throughout history in Asia until 1990s. The earliest available strain of GI was collected in Cambodia in 1967, and GI remained undetected for 10 years until another strain was identified in southwest China (Yunnan Province) in 1977 [28]. JEV strains were almost grouped into GIII before 2000 in China, but thereafter, the proportion of GI strains increased and became the dominant one in recent decades [29]. In the past few years, multiple reports have indicated a similar phenomenon in a number of Asian countries, such as Thailand, Korea, Japan, Malaysia, Vietnam, and India [28]. The mechanism of the genotype replacement had remained unknown until now. However, a viral multiplication experiment indicated a selective advantage of GI viruses for increased multiplicative ability in mosquito cells [28]. There is no firm evidence that different JEV genotypes circulating differ in their virulence [7].

In eastern China, the earliest strain of GI was collected from mosquito specimens in Shanghai in 2001. Subsequently, GI JEV strains had been identified in most other provinces. Genotype distribution data revealed that GI was gradually replacing GIII as the dominant genotype in the region (Table 6). The present study also indicated that Zhejiang JEV strains had changed from GIII to GI during the past decades, though the exact year was unknown due to a gap in surveillance activities for 24 years. According to a phylogeographic reconstruction study by Gao X et al, dispersal of GI lineage into Zhejiang was estimated to be in 2000 [30]. Similarly, several studies estimated the most recent common ancestor age of JEV and indicated that GI, as the youngest genotype, began to replace GIII approximately 20 years ago[9,31].

**Table 6:** Genotype distribution of Japanese encephalitis virus isolation in eastern China.

Isolate time (Year)	SH	JS	ZJ	SD	JX	FJ	AH	TW
1950s						III		III
1965								III
1972								III
1982			III					
1983			III					III
1985								III
1986								III
1987	III							III
1990								III
1994								III
1997								III
1998								III
2001	I							
2002						III		III
2003	I					Both		III
2004	III							
2005	I					III		III
2006	III							III
2007	I		I			III		III
2008	III	III		I		I		Both
2009		Both	I		I			Both
2010			I	I		I		Both
2011								I
2012			I					Both
2013			I	I				
2014			I					

Note: I, genotype I; III, genotype III; Both, genotype I and III were isolated in the same year. The genotype information of the strains referred to [5,8,29,32,33] and GenBank. The source of the strains might be samples from vectors, host animals or humans. Chinese provinces: SH, Shanghai; JS, Jiangsu; ZJ, Zhejiang; SD, Shandong; JX, Jiangxi; FJ, Fujian; AH, Anhui; TW, Taiwan.

The present work has some limitations. First, the sero study was confined to small local areas which might not reflect the provincial population immunity level. The conclusion of neutralizing antibody dynamic was not based on a follow-up design. Second, we did not adopt any PCR methods in phylogenetic analysis. It is likely that some pools of mosquitoes could negative for virus isolation but yielded amplified nucleic acids for analysis. Last, in the absence of 24 years interim, it is hard to come to a conclusion about a particular year when the genotype replacement event occurred in Zhejiang Province.

In conclusion, this study discovered that JE neutralizing antibody positive rate increases with age over 10 years, likely reflecting natural infection (in the unvaccinated) and natural boosting of immunity through exposure to wild virus (in the vaccinated). JE seropositivity rates were quite low in <35 years old age groups in Zhejiang Province. Waning of neutralizing antibody after live attenuated vaccine immunization was observed, but the clinical significance of waning antibody should be further investigated. Selection of different JEV strains in the neutralizing assay had considerable influence on antibody titer. JEV strains isolated in the recent years were all grouped into GI in Zhejiang Province, while those isolated in the 1980s belonged to GIII. On important amino acid sites related to antigenicity, there was no divergence between the Zhejiang JEV strains and SA14-14-2 strain. Both the peripheral antibody response and genetic characterization indicate that current live attenuated JE vaccine conferred equal neutralizing potency against GI or GIII wild strains. Although the chance of exposure to wild JEV has reduced, the virus is still regularly being isolated in nature. Therefore, it is necessary to implement immunization programs for children continually and to conduct surveillance activity periodically.

## Supporting Information

S1 Supplementary Data Microseroneutralization test results. (XLS)

S2 Supplementary Data Concise information of JEV strains isolated in Zhejiang Province. (PDF)

S1 Checklist Strobe Checklist. (DOC)
