# Supplementary material for: Sero-Molecular Epidemiology of Japanese Encephalitis in Zhejiang, an Eastern Province of China
Source: PLoS Negl Trop Dis. 2016 Aug 25;10(8):e0004936. doi: 10.1371/journal.pntd.0004936 (PMC4999095; doi:10.1371/journal.pntd.0004936)
Supplement: S2 Supplementary Data — (PDF) [file pntd.0004936.s002.pdf]

# Supporting Information

## S2

**Concise information of 28 strains of Japanese encephalitis virus isolated from Zhejiang**

| Strain | year | Source | Genotype | Accession no. |
|---|---|---|---|---|
| ZJ82-2 | 1982 | *Culex tritaeniorhynchus* | III | KJ000038 |
| ZJ82-6 | 1982 | *Culex tritaeniorhynchus* | III | KJ000039 |
| ZJ83-8 | 1983 | *Culex tritaeniorhynchus* | III | KJ000040 |
| ZJ83-13 | 1983 | *Culex tritaeniorhynchus* | III | KJ000041 |
| ZJ83-14 | 1983 | *Culex pipiens pallens* | III | KJ000042 |
| XJ69 | 2007 | *Culex tritaeniorhynchus* | I | EU258742 |
| XJP613 | 2007 | *Culex tritaeniorhynchus* | I | EU258741 |
| JX61 | 2008 | pig serum | I | GU556217 |
| ZJ09-52 | 2009 | *Culex tritaeniorhynchus* | I | JN216865 |
| ZJ109-08 | 2009 | *Culex tritaeniorhynchus* | I | JN216866 |
| ZJ10-07 | 2010 | *Culex tritaeniorhynchus* | I | JN216867 |
| ZJ10-10 | 2010 | *Culex tritaeniorhynchus* | I | JN216868 |
| ZJ10-45 | 2010 | *Culex tritaeniorhynchus* | I | JN216870 |
| ZJ12-03 | 2012 | *Culex tritaeniorhynchus* | I | KJ000029 |
| ZJ12-04 | 2012 | *Culex tritaeniorhynchus* | I | KJ000030 |
| ZJ12-06 | 2012 | *Culex tritaeniorhynchus* | I | KJ000031 |
| ZJ12-07 | 2012 | *Culex tritaeniorhynchus* | I | KJ000032 |
| ZJ13-02 | 2013 | *Culex tritaeniorhynchus* | I | KM079081 |
| ZJ13-03 | 2013 | *Culex tritaeniorhynchus* | I | KM079082 |
| ZJ13-06 | 2013 | *Culex tritaeniorhynchus* | I | KJ000033 |
| ZJ13-07 | 2013 | *Culex tritaeniorhynchus* | I | KJ000034 |
| ZJ13-09 | 2013 | *Anopheles sinensis* | I | KJ000035 |
| ZJ13-10 | 2013 | *Culex tritaeniorhynchus* | I | KJ000036 |
| ZJ13-11 | 2013 | *Culex tritaeniorhynchus* | I | KJ000037 |
| ZJ13-28 | 2013 | *Culex tritaeniorhynchus* | I | KM079084 |
| ZJ13-33 | 2013 | *Anopheles sinensis* | I | KM079085 |
| ZJ14-09 | 2014 | *Culex tritaeniorhynchus* | I | KM576776 |
| ZJ14-10 | 2014 | *Culex tritaeniorhynchus* | I | KM576777 |
